# Supplementary material for: A bioinspired surface tension-driven route toward programmed cellular ceramics
Source: Nat Commun. 2024 Jun 12;15:5030. doi: 10.1038/s41467-024-49345-3 (PMC11169415; doi:10.1038/s41467-024-49345-3)
Supplement: Supplementary file 1 — Supplementary Information [file 41467_2024_49345_MOESM1_ESM.pdf]

Supplementary Materials for

• A Bioinspired Surface Tension-driven Route toward Programmed Cellular Ceramics

Ying Hong,[1†] Shiyuan Liu,[1†] Xiaodan Yang,[1, 2†] Wang Hong,[3] Yao Shan,[1, 2] Biao Wang,[4] Zhuomin Zhang,[1, 2] Xiaodong Yan,[1, 2] Weikang Lin, [1, 2] Xuemu Li,[1] Zehua Peng,[1, 2] Xiaote Xu,[1, 2] Zhengbao Yang[1]*

*Corresponding author. Email: zbyang@ust.hk

This PDF file includes:

Supplementary Text 1 to 3
Supplementary Figure 1 to 22
Supplementary Table

Other Supplementary Materials for this manuscript include the following:
Supplementary Movie 1 to 3

Supplementary Text

1. **Numerical calculation of the design principle of the cellular lattices**

Design principle of a unit cell

As the unit cell moves from the prepared solution (fluid 1) to air (fluid 2), the solution is captured in the solid frame (solid 3) only when $E_1<E_2$, where E_1 and E_2 are the interfacial free energy for solution capture and solution collapse, respectively. The total interfacial free energy E_1 and E_2 can be expressed as[25],

$$E_1=S_{23}\sigma_{23}+S\sigma_{12}+S_{13}\sigma_{13}+S_{12}\sigma_{12} \tag{1}$$

$$E_2=S_3\sigma_{23}+S\sigma_{12} \tag{2}$$

where σ_{23}, σ_{12}, σ_{13} are the interfacial tension of fluid 2-solid 3, fluid 1-fluid 2 and fluid 1-solid 3 respectively, S is the initial interface area of fluid 1 and fluid 2, and S_{12} is the created interface of fluid 1 and fluid 2. S_3 is the total surface area of the solid frame and satisfy $S_3=S_{13}+S_{23}$, where S_{13} and S_{23} are the contact area of fluid 1-solid 3 and fluid 2-solid 3, respectively. Given the contact angle of fluid 1 on solid 3 in fluid 2 shown as Fig. 3b, the Young equation is

$$\sigma_{23}=\sigma_{13}+\sigma_{12}\cos\theta_{123} \tag{3}$$

where θ_{123} is the contact angle of fluid 1 on solid 3 in fluid 2 (Fig. 3b). Combining Equations 1-3, the equation $E_1<E_2$ can be converted into

$$S_{12}-S_{13}\cos\theta_{123}<0 \tag{4}$$

To better elaborate the detailed design principle, we take a cube cell with a frame length of a and a frame radius of r for example (Fig. 3a). We define α as the ratio of S_{13} to S_3, and it also satisfy

$$\alpha=S_{13}/S_3=k/2\pi \tag{5}$$

where k is the central angle corresponding to the interface arc of fluid 1-solid 3. So S_{13} can be calculated as

$$S_{13}=2\pi\alpha(a-2r)\times 12 \tag{6}$$

And S_{12} can be calculated as

$$S_{12}\geq 6(a-2r)^2 \tag{7}$$

Combining Equations 4, 6 and 7, and the geometry requirement ($a > 2r$), we obtain

$$2<a/r<4\pi\alpha\cos\theta_{123}+2 \tag{8}$$

In addition, the size of the created fluid interface must be within the capillary length, a characteristic length scale for the fluid subject to a body force from gravity and a surface force due to surface tension, which means

$$a-2r<\sqrt{\sigma_{12}/\Delta\rho_{12}g} \tag{9}$$

where $\Delta\rho_{12}$ is the density difference between fluid 1 and fluid 2, and g is the gravitational acceleration.

Therefore, equations 8 and 9 summarize the geometrical requirements for the unit cube cell. Given that θ_{123}=20°, σ_{12}=60.6 mN/m, and $\Delta\rho_{12}=2.3\times10^3$ kg/m^3. The α=0.98 can be calculated by the finite element analysis (Fig. 2c, Supplementary Fig. 15 and Movie 2), the equation 8 and 9 are specified into

$$2.0 < a/r < 14.3 \tag{10}$$

$$a - 2r < 1.64 \text{ mm} \tag{11}$$

As shown in Fig. 3d, the blue region represents the safe area with appropriate frame length and radius to successfully create stable liquid interface. Further experiments are also in good agreement with the theoretical analysis (Fig. 3d and Supplementary Fig. 16).

Design principle of a unit column

Besides the frame length and radius of the unit cell, the height of the unit column is another parameter to be considered under the influence of gravity. According to Jurin's law, the liquid height in the unit column can be estimated by the equilibrium of surface tension force F_{st} and gravitational force F_g, which is $F_{st}=F_g$. The surface tension force is estimated assuming an infinitesimally thin planar gas-liquid interface across the structure, which is given by[1]

$$F_{st}=\sigma_{12}\cos\theta_{123}s \quad (12)$$

where s is the liquid-solid contact perimeter. The gravitational force depending on the liquid volume is can be calculated by

$$F_g=\rho gha^2(1-\varphi) \quad (13)$$

Where ρ is the liquid density, h is the liquid height, and φ is the volume ratio of the solid frame to the unit cell. Combining Equations 12 and 13, we obtain

$$h=\frac{\sigma_{12}\cos\theta_{123}s}{\rho ga^2(1-\varphi)} \quad (14)$$

The liquid-solid contact perimeter varies with the frame radius and the top plane position of the liquid, and always reaches a maximum when contacting with the top frame of the cell. According to Equation 14, under the equilibrium state, a larger contact perimeter s results in a larger liquid height h. In a cube cell, there are mainly two states for the contact perimeter (red dash line) according to the top plane position (liquid height) of the liquid (Supplementary Fig. 17a). Here we define $s^*=s/2\pi r$ to get a dimensionless parameter. Supplementary Fig. 17b summarizes the relation between liquid height and the contact perimeter s^* in a unit cell as the radius increases from 0.1 mm to 0.25 mm. The contact perimeter s^* in state ii is larger than that in state i so that the surface tension force in state ii is also larger than that in state i. Fig. 3e summarizes the relation between liquid height and the maximum contact perimeter in a unit column as the radius increases from 0.1 mm (red dash line) to 0.25 mm (purple dash line). A relation between the liquid height h and the contact perimeter s^* with corresponding radii from gravitational force is also given in Fig. 3e (solid line) and Supplementary Fig. 17c (dash line) according to Equation 14. By solving Equation 14, the equilibrium point (star icons in Fig. 3e) of surface tension force F_{st} and gravitational force F_g is obtained. The increasing frame radius leads to a higher maximum contact perimeter and volume ratio φ, corresponding to a higher surface tension force and lower liquid gravity. As a result, the equilibrium point shifts with increasing frame radius, contributing to a higher liquid height (Fig. 3f).

Design principle of an architected lattice

When considering the whole architected lattice, in which the precursor solution is assembled with a programmed arrangement, both of the successful capture in parts of the unit cells and the failed capture in other parts are required. Taking the architected lattices depicted in Fig. 3g for example, the unit cells with a length of a should be filled with the precursor solution while it is the opposite for the cells with a length of i) $2a$, ii) $3a$, and iii) $4a$. As a result, the constraints,

shown as the yellow region in Fig. 3d, consisting of 7.15 < a/r < 14.3 (blue dash line and blue solid line) and 0.82 mm < $a - 2r$ < 1.64 mm (red dash line and red solid line), are required to form a programmed arrangement with an interval length of i) $2a$ depicted in Fig. 3g. The constraints of 4.77 < a/r < 14.3 and 0.55 mm < $a - 2r$ < 1.64 mm are required to form a programmed arrangement with an interval length of ii) $3a$. The constraints of 3.58 < a/r < 14.3 and 0.41 mm < $a - 2r$ < 1.64 mm are required to form a programmed arrangement with an interval length of iii) $4a$. FE analysis is further utilized to verify the calculated and experimental results, as shown in Fig. 3h, Supplementary Fig. 18 and Movie 3.

2. Surfactants addition and plasma treatment

The addition of surfactants in the precursor solution and the plasma treatment on the organic lattices also benefit the decrease of contact angle to some extent. Under the condition of 2 mM concentration of the precursor solution, the contact angle is 20° while the addition of 1 wt.% surfactants in the precursor solution reduces the contact angle to 13° and a plasma treatment (2 min) on the organic lattices reduces it to 15°. Considering that a contact angle of 20° is enough to form a lyophilic condition (α is 0.98 at the contact angle of 20°, close to its maximum), these factors are not considered in the following experiments from the perspective of simplification of experimental procedures.

3. Electrical responses of the STATS manufactured piezoelectric ceramics

The electrical responses of the piezoelectric materials under mechanical deformation originate from the direct piezoelectric effect. Under compressive pressure, the d_{33} constants of the cellular PZT ceramics are quantified by the ratio of the applied load F_N and the generated charge Q, which is given by $d_{33}=Q/F_N$. And the g_{33} constants are further quantified by $g_{33}=d_{33}/\varepsilon_{33}$. The effective permittivity ε_{33} can be calculated by $\varepsilon_{33}=Cl/A$, where C is the capacitance of the cellular PZT ceramics, l is the distance between the electrodes and A is the cross-section area of the cellular PZT ceramics. In addition, the generated charge Q can be expressed by $Q=CV_{out}$, and the applied load F_N can be expressed by $F_N=PA$, where V_{out} is the open-circuit output voltage and P is the applied pressure. Combining these equations, the open-circuit output voltage can be given by

$$\frac{V_{out}}{P}=g_{33}\, l \tag{15}$$

As a result, the open-circuit output voltage is linearly correlated with the applied pressure, and a larger g_{33} constant leads to an increasing output voltage.

The calculated open-circuit output voltage according to Equation 15 should be under quasi-static state, usually larger than the output voltage measured in practical application. Nevertheless, this equation is helpful to understand that a larger g_{33} constant leads to an increasing pressure sensitivity dV_{out}/dP.

Supplementary Figures

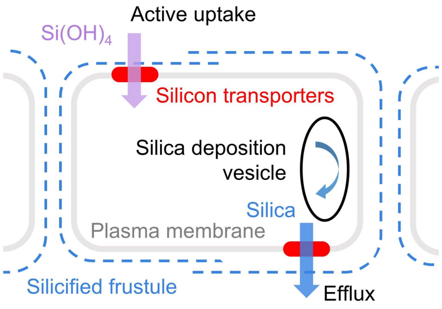

Supplementary Fig. 1. The biomineralization of silica frustule, including the transport of silicic acid through silicon transporters (SITs) and the silification process in silica deposition vesicle (SDV). During the biomineralization of silica frustule, Si (primarily in the form of silicic acid, $Si(OH)_4$) is first actively taken from seawater into the cell by the SITs. Upon uptake, silicon is transported to the SDV, where silification process takes place. And then, the precipitated and polymerized silica is transported through the plasma membrane to form silicified frustule.

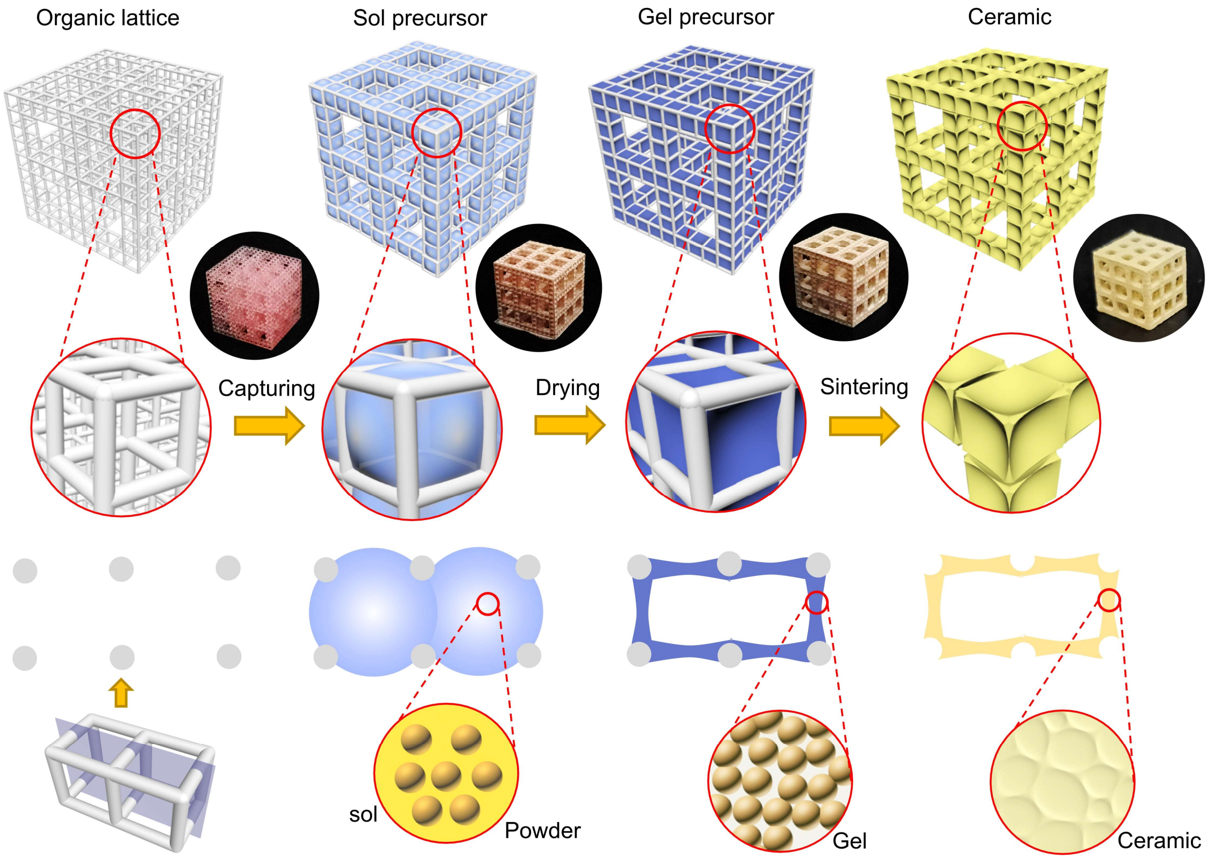

Supplementary Fig. 2. Illustration of the surface-tension-assisted two-step (STATS) manufacturing process, including precursor solution capture, sol-gel transition, and high-temperature sintering. Insets show the optical images of samples during the manufacturing. The fabricated organic lattices are first immersed in prepared precursor solution. Once we remove the lattices from the solution to air, part of the precursor solution is captured in the lattices with a programmed arrangement by the surface tension. After drying, a sol-gel transition occurs, leading to the shrinkage of precursor solution. The obtained gel precursor forms a cellular shell structure, packaging the organic lattice. The final sintering process at high temperature transforms the gel precursor into compact ceramics.

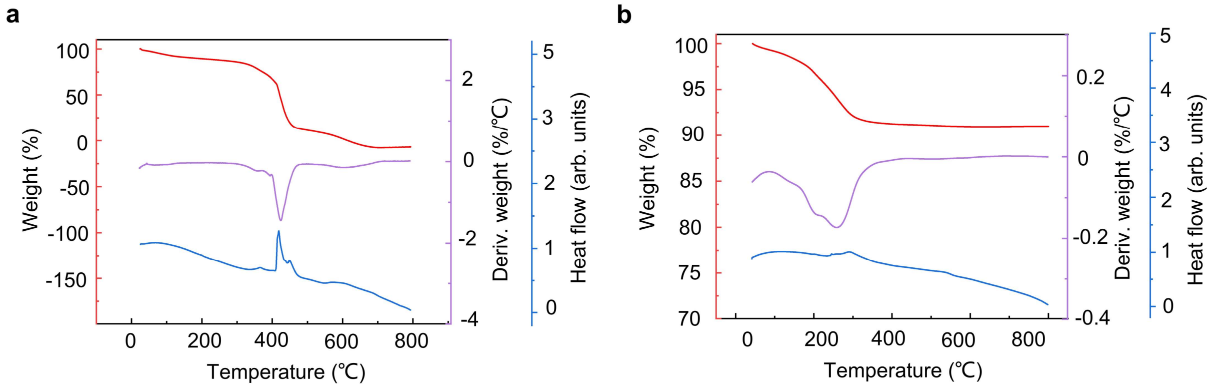

Supplementary Fig. 3. Differential scanning calorimetry (DSC) and thermogravimetric (TG) curves of **a**, the 3D-printed organic lattice and **b**, the dried gel precursor during the sintering process.

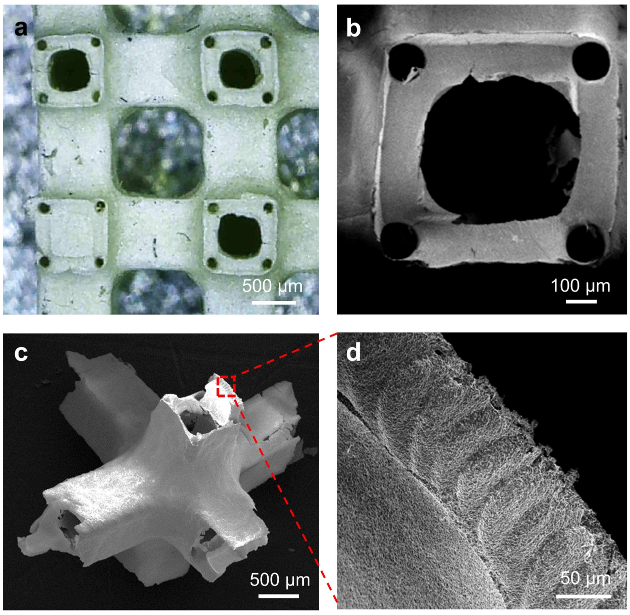

Supplementary Fig. 4. **a**, Optical image and **b** & **c**, Scanning electron microscopic (SEM) images of the cellular ceramics, showing the cell-based globally cellular shell structure. **d**, Enlarged SEM image showing the layer-by-layer characteristic of the interface with the 3D-printed organic lattice.

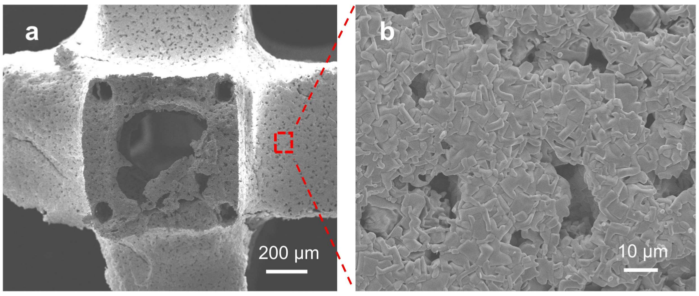

Supplementary Fig.5. SEM images of the lead zirconate titanate (PZT) cellular ceramics prepared from the conventional powder-binder-water suspensions, showing a lower quality with more defects.

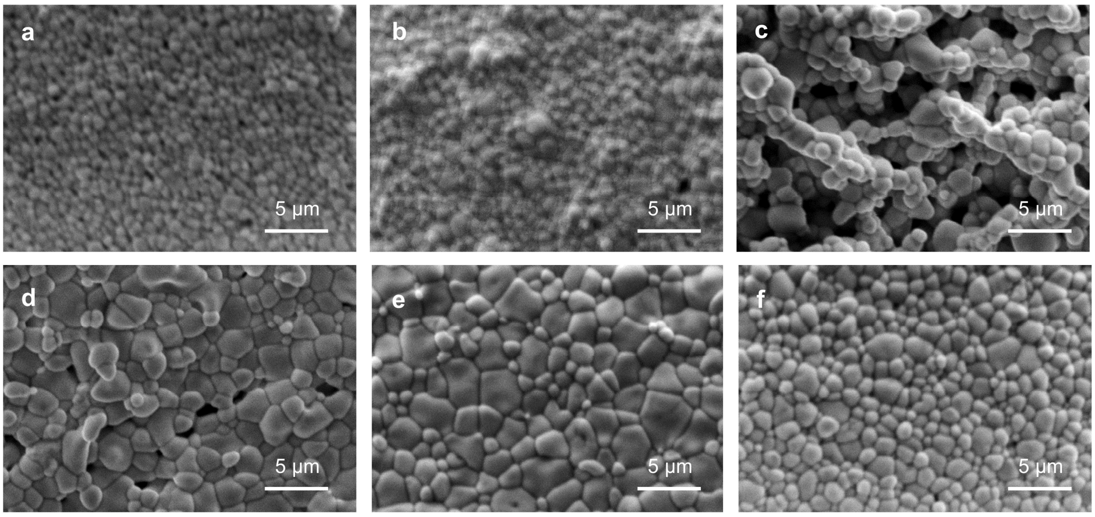

Supplementary Fig. 6. SEM images of the sintered ceramics depending on the mass ratio of the powder to sol solution in the preparation of precursor suspension from **a**, 1:8, **b**, 1:4, **c**, 1:2, **d**, 1:1, **e**, 2:1, to **f**, 4:1. The concentration of sol solution is 2 mM.

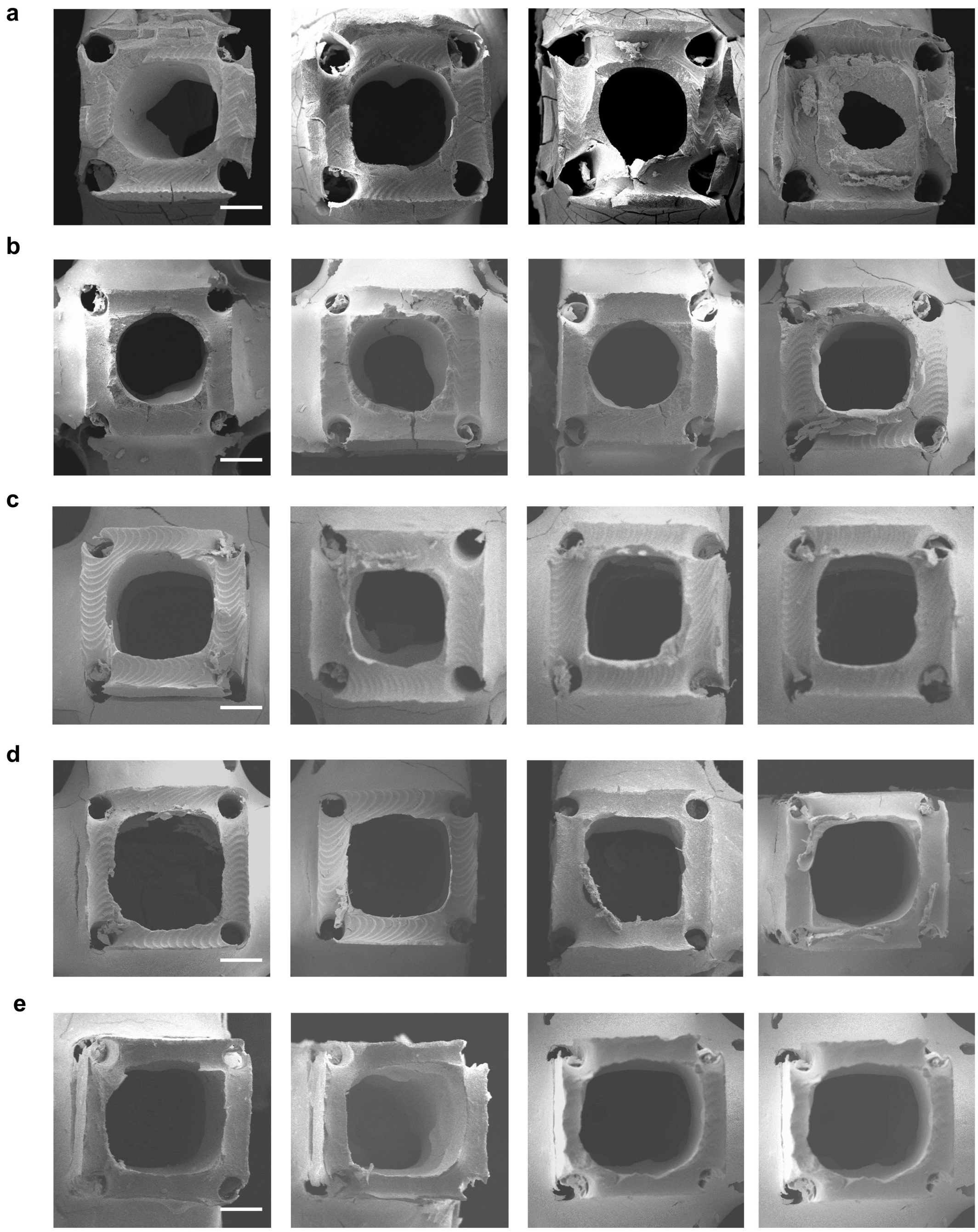

Supplementary Fig. 7. SEM images of the sintered PZT cellular ceramics prepared from the precursor solution with various concentrations from **a**, 5 mM; **b**, 4 mM; **c**, 3mM; **d**, 2 mM; **e**, 1 mM. The organic lattice has a 1 mm frame length and a 100 μm frame radius.

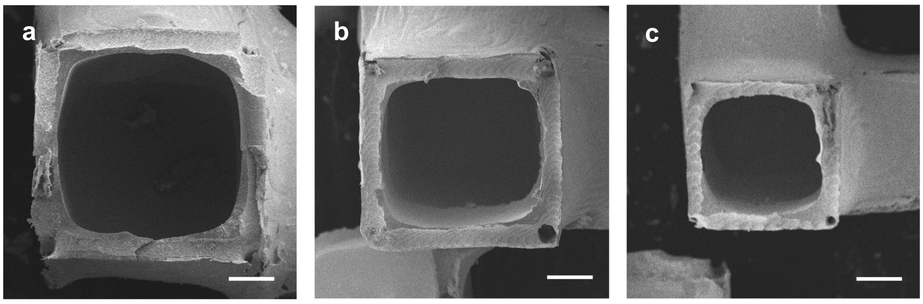

Supplementary Fig. 8. **a**, SEM image of the sintered cellular ceramics with a chamber length of 0.99 mm and a shell thickness of 90 μm, at a frame length of 1.5 mm and a frame radius of 100 μm of the organic lattice. **b**, SEM image of the sintered cellular ceramics with a chamber length of 0.83 mm and a shell thickness of 90 μm, at a frame length of 1.25 mm and a frame radius of 100 μm of the organic lattice. **c**, SEM image of the sintered cellular ceramics with a chamber length of 0.65 mm and a shell thickness of 65 μm, at a frame length of 1 mm and a frame radius of 75 μm of the organic lattice. The concentration of precursor solution is 1 mM. Scale bar, 200 μm.

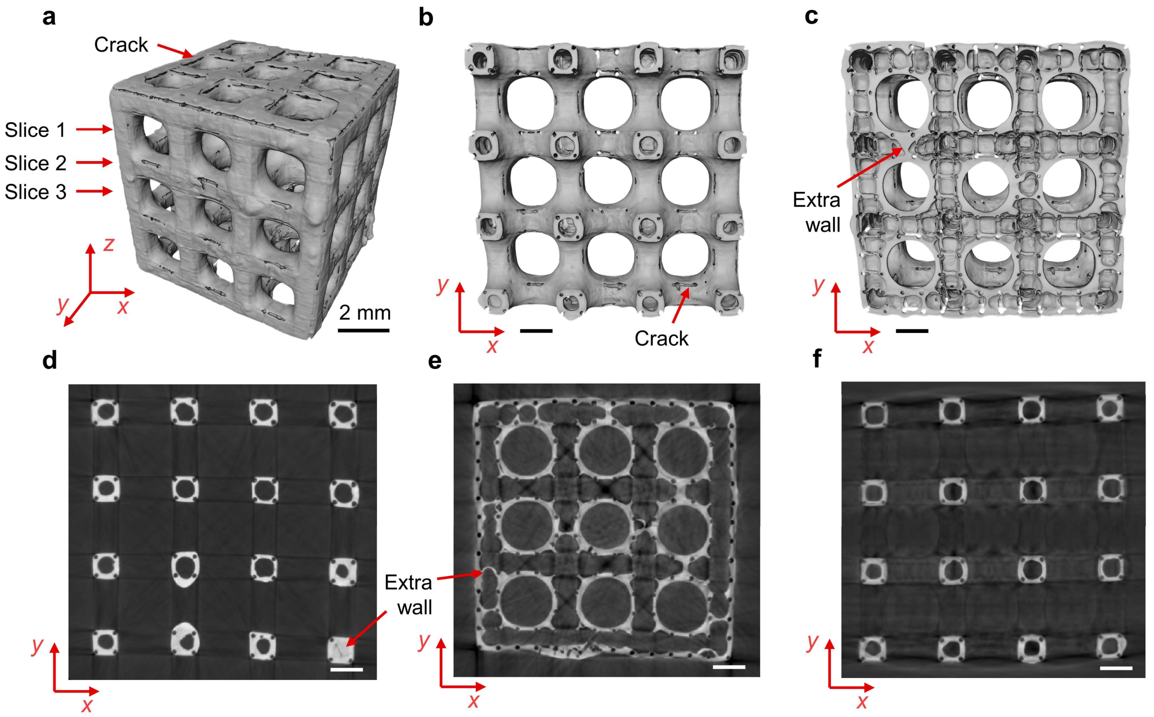

Supplementary Fig. 9. **X-ray computed microtomographic (micro-CT) images of the sintered cellular PZT ceramics.** **a**, Reconstructed model of the ceramic sample. **b&c**, Cross-section view from **b**, slice 1 and **c**, slice 2. Scale bar, 1 mm. **d, e, &f**, Single slices from **d**, slice 1 and **e**, slice 2 and **f**, slice 3. The micro-CT images clearly show the globally cellular shell structure of the sintered ceramics with a high quality. There are mainly two types of defects, including the cracks existing in the interface between the ceramic shell and the removed organic lattices, and the extra ceramic walls between the unit cells. Scale bar, 1 mm.

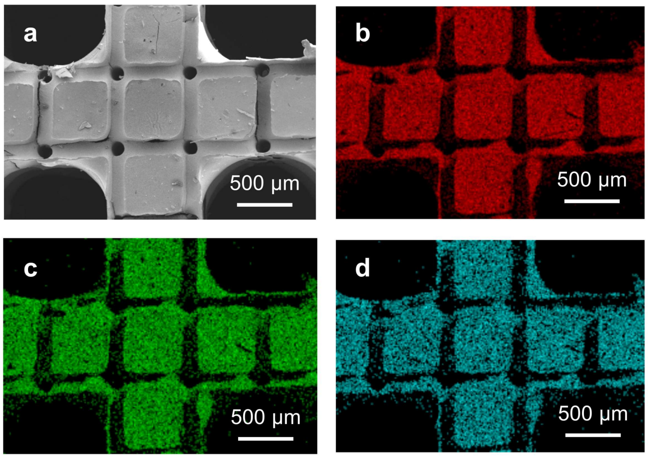

Supplementary Fig. 10. Energy dispersive spectroscopic (EDS) mapping of the STATS manufactured cellular PZT ceramics, showing that the elements of Pb, Zr, and Ti have a homogeneous distribution in the architected ceramics.

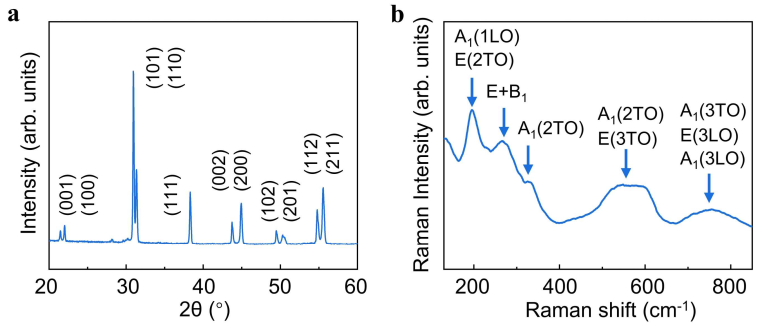

Supplementary Fig. 11. **a**, X-ray diffraction (XRD) patterns and **b**, Raman spectrum of the STATS manufactured PZT ceramics.

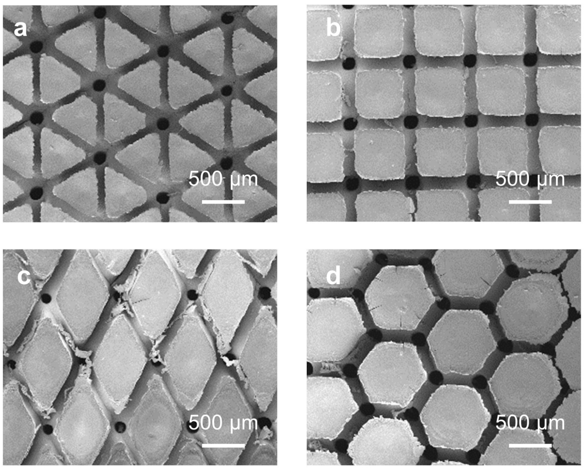

Supplementary Fig. 12. SEM images of the manufactured cellular ceramics with various cell geometries, including **a**, triangle, **b**, square, **c**, diamond and **d**, Hexagon.

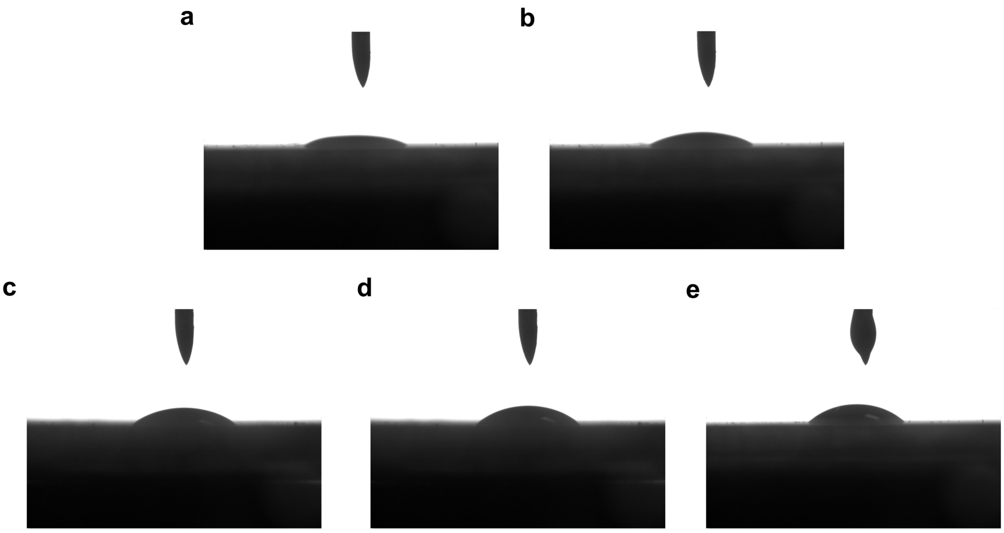

Supplementary Fig. 13. Contact angle of the precursor solution on the resin plate in air depending on the solution concentration. **a**, 15.2° at 1 mM. **b**, 20.0° at 2 mM. **c**, 29.6° at 3 mM. **d**, 35.7° at 4 mM. **e**, 44.0° at 5 mM. The contact angle increases, accompanied by the increasing concentration, but all within 90°.

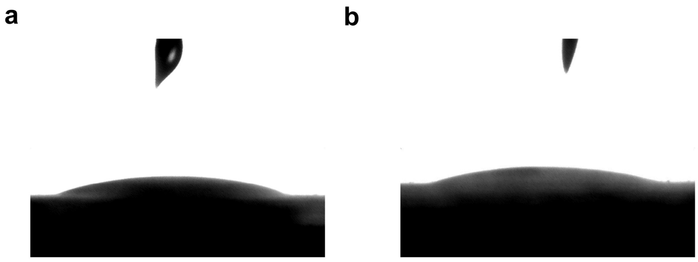

Supplementary Fig. 14. Contact angle of the precursor solution on the resin plate in air. **a**, Contact angle (15°) after a plasma treatment (2 min) on the resin plate. **b**, Contact angle (13°) after the addition of 1 wt.% surfactants in the precursor solution.

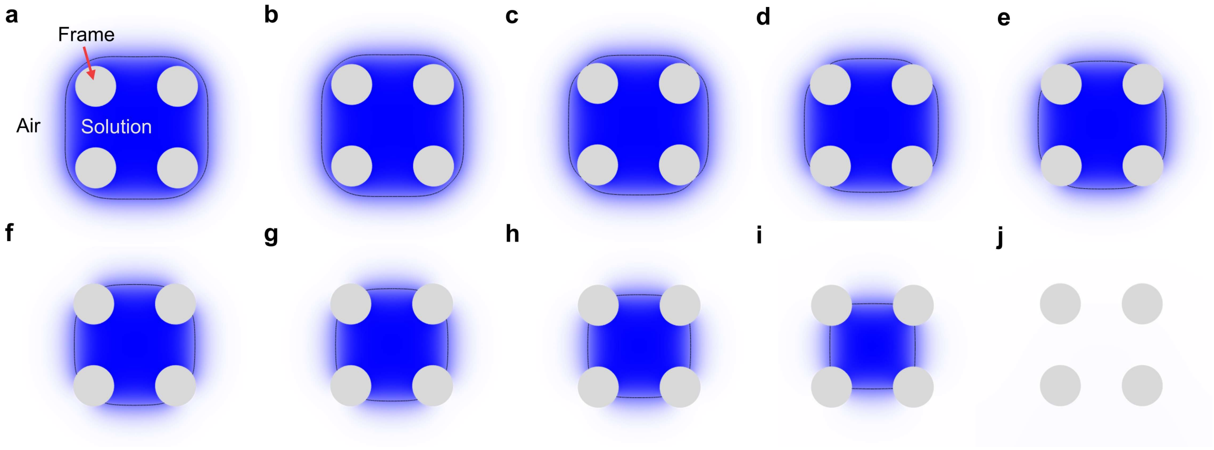

Supplementary Fig. 15. The finite element simulation results for solution-in-air interface creation using cube frames with different contact angle from **a**, 0° to **j**, 90°. The calculated α decreases from 1 to 0 when the contact angle increases from 0° to 90°.

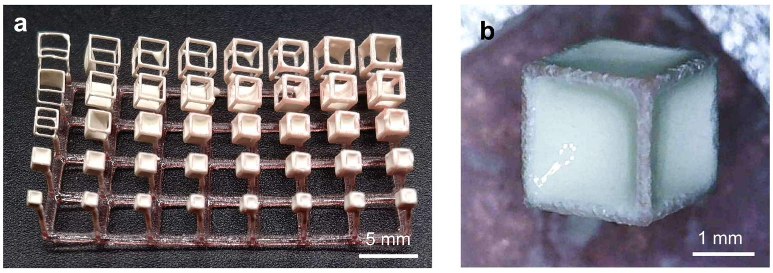

Supplementary Fig. 16. a, Optical image of the liquid capture results with various frame lengths from 1 mm to 3 mm and radii from 0.075 mm to 0.25 mm. **b**, Enlarged optical image of the liquid capture results with a frame length of 2 mm and a radius of 0.1 mm.

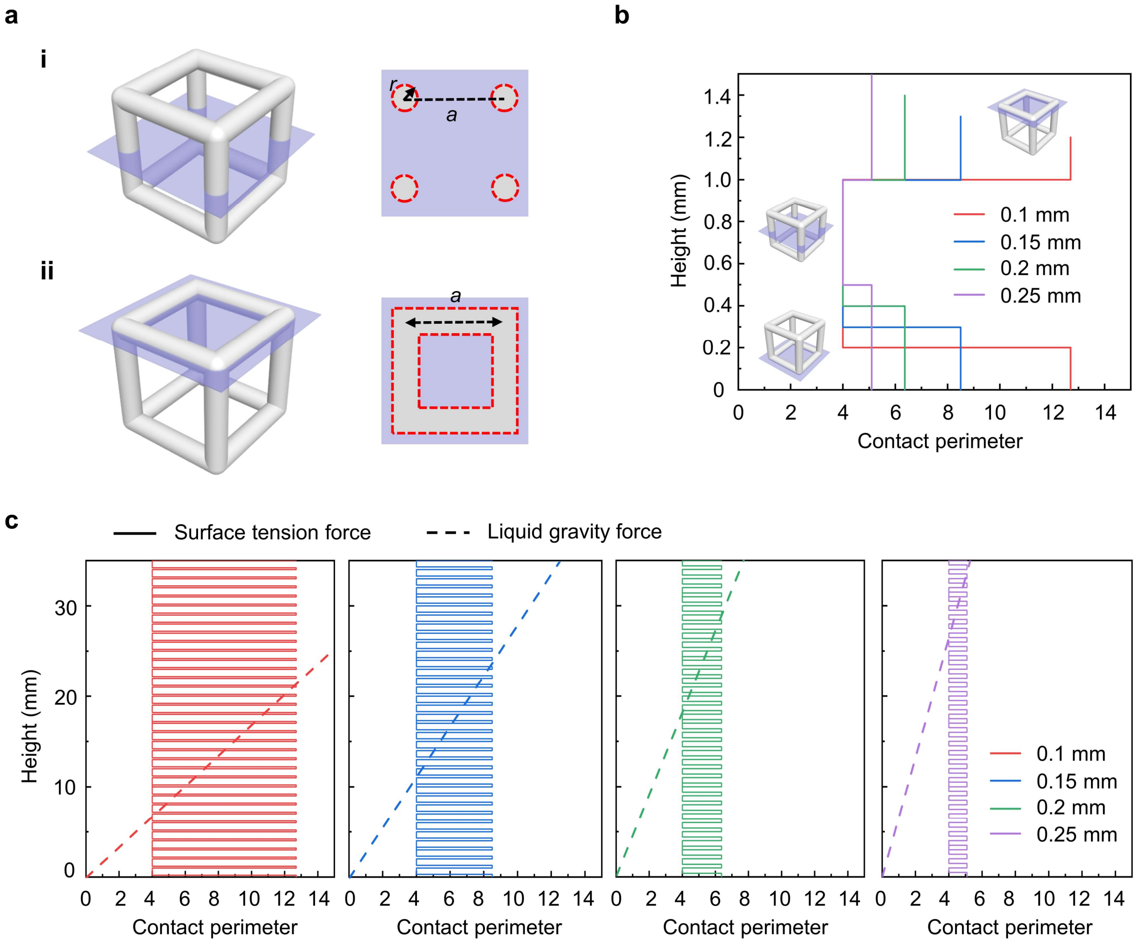

Supplementary Fig. 17. **a**, Two states for the contact perimeter (red dash line) according to the top plane position (liquid height) of the liquid. **b**, The relation between liquid height and the contact perimeter s^* in a unit cell as the radius increases from 0.1 mm to 0.25 mm. The contact perimeter s^* in state ii is larger than that in state i so that the surface tension force in state ii is also larger than that in state i. **c**, Solid line presents the relation between liquid height h and contact perimeter s^* in a unit column as the radius increases from 0.1 mm (red solid line) to 0.25 mm (purple solid line). Dash line presents the relation between the liquid height h and the contact perimeter s^* with corresponding radii from gravitational force.

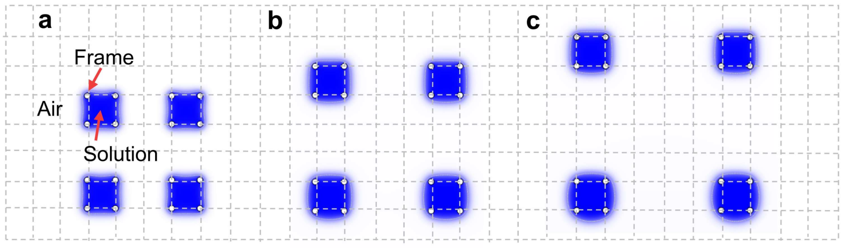

Supplementary Fig. 18. Finite element (FE) analysis of the liquid arrangement for the architected lattice with an interval length of **a**, 2a, **b**, 3a, and **c**, 4a.

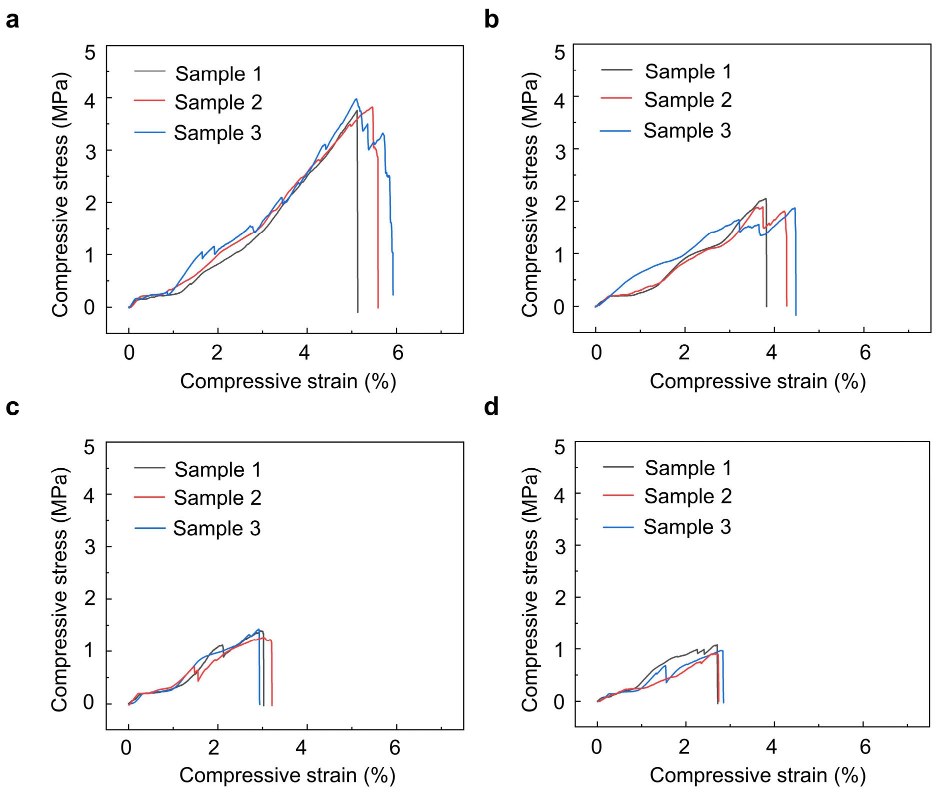

Supplementary Fig. 19. The compressive stress-strain curves of the prepared cellular PZT ceramics with a relative density of **a**, 0.34; **b**, 0.27; **c**, 0.21; and **d**, 0.13.

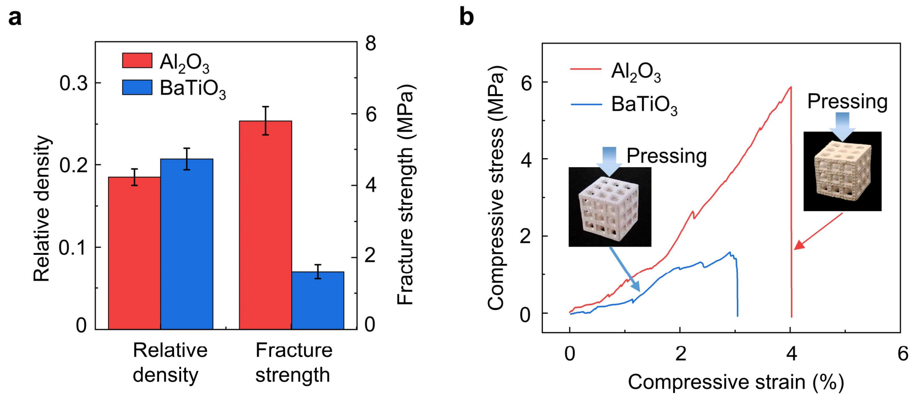

Supplementary Fig. 20. **a**, The relative densities and fracture strength of the prepared Al_2O_3 and $BaTiO_3$ cellular ceramics. Error bars are standard deviation and include 5 independent measurements for each composition. **b**, The compressive stress-strain curves of the prepared Al_2O_3 and $BaTiO_3$ cellular piezoceramics.

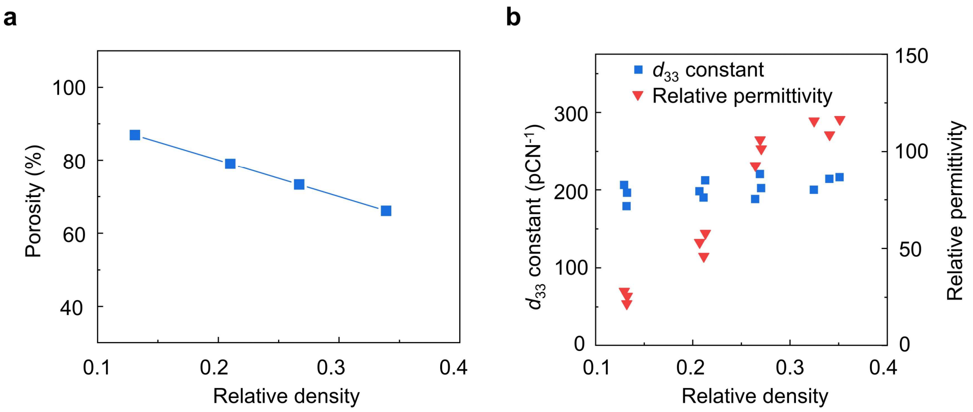

Supplementary Fig. 21. **a**, The corresponding porosity to the relative density, in which the relative density of 0.13, 0.21, 0.27, and 0.34 corresponds to a porosity of 0.980, 0.963, 0.951 and 0.931, respectively. **b**, The effective d_{33} and relative permittivity of the prepared cellular piezoceramics with a relative density of 0.13, 0.21, 0.27 and 0.34.

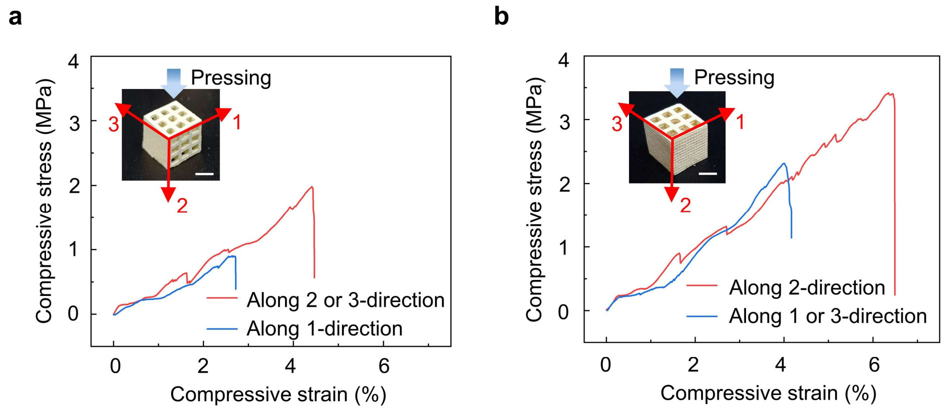

Supplementary Fig. 22. The compressive stress-strain curves of the prepared cellular PZT ceramics with **a**, Type ii configuration and **b**, Type iii configuration.

Supplementary Table

Supplementary Table 1. Comparison of the proposed STATS process with the DIW, SLA, DLP, and SLS.

3D printing method	References	Materials	Effective constituent loading	Fabrication speed	Accessible feature size
DIW	Dutto *et al.*[35]	Clay	20-50 wt%	5.6-62.9 mm s^{-1}	1.6-6.3 mm lateral and 2-4.5 mm height
	Maurath *et al.*[36]	Al_2O_3	23-31 vol%	42-55 mm s^{-1}	0.2-0.62 mm lateral and 2-4.5 mm height
	Smay *et al.*[37]	PZT	47 vol%	6 mm s^{-1}	0.2-0.4 mm lateral and 0.16-0.32 mm height
	Menne *et al.*[38]	$BaTiO_3$	31 vol%	10 mm s^{-1}	0.07-0.15 mm lateral and 0.12 mm height
SLA	Griffith *et al.*[39]	Al_2O_3	40-50 vol%	NA	0.2-0.4 mm height
	Liu *et al.*[40]	ZrO_2-Al_2O_3	46.8 vol%	8 m s^{-1}	0.04 mm lateral
	Chen *et al.*[41]	$BaTiO_3$	70 wt%	NA	0.02 mm lateral and 0.05 mm height
DLP	Chen *et al.*[42]	$BaTiO_3$	40 vol%	NA	0.01 mm height
	Komissarenko *et al.*[43]	ZrO_2	33 vol%	5 s per layer	0.05 mm height
	Varghese *et al.*[44]	Al_2O_3	45-55 wt%	5-7 s per layer	0.045 mm lateral and 0.025-0.05 mm height
SLS	Bertrand *et al.*[45]	ZrO_2-Y_2O_3	NA	1.25-2 mm s^{-1}	0.52-1 mm lateral
	Zhang *et al.*[46]	$BaTiO_3$	NA	0.5 mm s^{-1}	1 mm lateral
STATS process	This work	PZT	> 90 wt% for dried gel	2 s per layer for organic lattice	0.1-0.2 mm shell thickness
