# Supplementary material for: A bioinspired surface tension-driven route toward programmed cellular ceramics
Source: Nat Commun. 2024 Jun 12;15:5030. doi: 10.1038/s41467-024-49345-3 (PMC11169415; doi:10.1038/s41467-024-49345-3)
Supplement: Supplementary file 3 — Description of Additional Supplementary Files [file 41467_2024_49345_MOESM3_ESM.pdf]

## Description of Additional Supplementary Files

File Name: Supplementary Movie 1

Description: Precursor solution captured in the architected lattice. The video shows capture process of the precursor solution in the architected lattice. The fabricated organic lattice is immersed in prepared precursor solution. Once we remove the lattices from the solution to air, part of the precursor solution is captured in the lattices with a programmed arrangement by the surface tension.

File Name: Supplementary Movie 2

Description: Finite element simulation of the interface creation. The video shows the finite element simulation results for solution-in-air interface creation using cube frames with different contact angle of 20°, 60°, 80° and 90°.

File Name: Supplementary Movie 3

Description: Finite element simulation of the liquid arrangement. The video shows the finite element analysis of the formation of the programmed liquid arrangement under gravity for the architected lattices with an interval length of 2a, 3a, and 4a.
